# Effects of Microwave Heating and Long-Term Aging on the Rheological and Chemical Properties of Recovered Bitumen

**DOI:** 10.3390/ma14247787

**Published:** 2021-12-16

**Authors:** Matías Fernández, Gustavo Canon, Sabine Leischner, Mrinali Rochlani, José Norambuena-Contreras, Alvaro González

**Affiliations:** 1 Department of Construction Engineering and Management, School of Engineering, Pontificia Universidad Católica de Chile, Santiago 7820436, Chile; mafernandez2@uc.cl; 2 Institute of Urban and Pavement Engineering, Technische Universität Dresden, 1062 Dresden, Germany; gustavo_adolfo.canon_falla@tu-dresden.de (G.C.); sabine.leischner@tu-dresden.de (S.L.); mrinali_rajkumar.rochlani@tu-dresden.de (M.R.); 3 LabMAT, Department of Civil and Environmental Engineering, University of Bío-Bío, Concepcion 4051381, Chile; jnorambuena@ubiobio.cl

**Keywords:** asphalt pavements, stone mastic asphalt, aged bitumen, self-healing asphalt, microwave heating technology, rheological and chemical properties

## Abstract

Microwave heating of asphalt pavement is a promising technique to reduce the maintenance and increase the service life of materials through self-healing of cracks. Previous studies have shown that microwave heating technology at high temperatures could damage the bitumen of asphalt mixture, which is an unwanted effect of the crack-healing technique. In this study, the effects of microwave heating and long-term aging on the rheological and chemical properties of recovered bitumen were quantified using a frequency sweep test and Fourier Transform Infrared Spectrometry analysis, respectively. The main results indicate that microwave heating has no significant effect on the aging performance of G* and δ for aged asphalt mixtures. However, for newer bitumens, the rheological properties G* and δ show minor changes after microwave heating was applied. Overall, this study confirms that microwave heating is a potential alternative for maintenance of asphalt pavements, without severely affecting the rheological and chemical properties of bitumen.

## 1. Introduction

Asphalt mixture is the most widely used material for pavement road construction because it provides good mechanical performance, economy, and construction advantages [1]. Despite its good properties as a road material, asphalt mixture deteriorates over time, with cracking and bitumen aging being the most common forms of damage [2]. Cracking is mainly caused by repetitive traffic loading and environmental factors that trigger bitumen aging [3]. The phenomenon of bitumen aging mainly consists of an oxidation process and polymeric degradation, which modifies the microstructure of bitumen [4]. The irreversible oxidation process is controlled by thermal reaction between oxygen molecules and the bitumen components, which alters its chemical features [4,5]. This type of aging occurs during the production, transportation, and laying of the mixture (short-term aging) at a very fast rate, and it continues during the service life of the pavement (long-term aging) [4,5]. During the oxidation process, the functional chemical groups of the bitumen, such as the carbonyl (C=O) and sulfoxide (S=O) groups, increase the overall polarity of the bitumen, which causes agglomeration among molecules due to increased physicochemical association [6]. As a result, the chemical changes reduce the viscoelastic properties of the bitumen, making bitumen stiffer until it becomes a brittle material and reduces its adhesion to aggregates [7]. The stiff and brittle bitumen causes the asphalt mixture to crack, which in turn reduces the pavement capability to withstand repeated traffic loads and shortens the pavement life [8].

In recent studies, microwave heating has been proposed as a promising technique to reduce the traditional maintenance and increase the service life of materials through self-healing of cracks [9,10,11]. During the heating process, microwave radiation applies alternating electromagnetic fields with a higher frequency, causing a change in the orientation of polar molecules, which result in internal friction and increase the material temperature [12]. In this way, at 30–70 °C, the bitumen in asphalt mixtures reduces its viscosity and begins to flow through microcracks [13]. When pavements cools to lower temperatures, the bitumen fills and seals the crack increases its viscosity, and the damage get healed [14]. This novel technique has proved that after one cycle of microwave heating of conventional asphalt, pavement achieves a 20% fatigue life extension [15]. To improve the electrical conductivity and thermal distribution of the asphalt mixtures with self-healing properties, metallic waste or steel wool fibers must be added to the asphalt matrix [11,16,17,18,19,20]. However, Gonzalez et al. [1] found that asphalt mixtures without metallic additives are also capable of healing their cracks by microwave heating; therefore, existing asphalt pavements could also be crack-healed through microwave heating.

The self-healing of asphalt mixture is a temperature-dependent phenomenon, and it is necessary to heat the bitumen for a sufficient time to reach an adequate viscosity change for healing [21]. Previous research has reported that the self-healing of asphalt mixtures by microwave heating can be achieved with a heating time of 40 s [22]. Norambuena-Contreras and Garcia [23] evaluated the surface temperature of dense asphalt mixtures with different percentages of metallic fibers for various heating times, observing that samples with 8% fibers reached 135 °C after 120 s of heating. In addition, Flores et al. [24] conducted a thermographic analysis to measure the temperature of Marshall specimens during microwave radiation heating. The results showed that, after 300 s of microwave heating, the specimens reached an average temperature of 120 °C. However, when the temperature is too high, it may decrease the healing level due to drainage of the bitumen under gravity.

Bitumen also tends to suffer more serious aging damage at higher temperatures [25], and the oxidation process of its components can be significantly accelerated, which could potentially decrease the durability of the mixtures. Additionally, temperature significantly influences the kinetics of aging, with those effects related to the bitumen. In general terms, the rate of oxidation doubles with each 10 °C rise in temperature above 100 °C [6]. Thus, microwave heating can age the bitumen in the asphalt mixture, which is an unwanted effect of the crack-healing technique, the healing capability of asphalt mix decrease as the aging level increases [26]. The influence of temperature on bitumen by effect of the microwave heating was investigated for the first time by Norambuena-Contreras and Garcia [23]. To do this, the authors carried out thermogravimetric analysis on virgin bitumen combined with microwave heating tests on asphalt mixture samples in a range of fibers amounts before and after several heating cycles. The main results proved that the temperature of the binder under microwave heating can be higher than the flash point temperature of bitumen [27]; consequently, microwave heating may damage the chemical structure of the binder used into the self-healing asphalt mixtures. However, this result has not been tested on mixtures without fibers, although González et al. [12] recently found promising crack-healing results on mixtures without fibers. Moreover, rheological and chemical tests have not been performed to evaluate bitumen aging on this type of mixtures.

Additionally, Wu et al. [28] investigated the effect of microwave heating on the physical properties of a bitumen 60/70 pen. The bitumen was heated to a target temperature of 150 °C and cooled to room temperature near 25 °C. The researchers measured the penetration, ductility, and softening point of the bitumen after one, three, and five microwave-heating cycles to evaluate the bitumen aging. The results showed, after five cycles of microwave heating, a reduction of 3.87% in penetration value, a 9.19% increase in softening point, and a 25.93% decrease in ductility. They found no clearly negative effect of microwave heating and concluded that the microwave heating causes slight aging in the bitumen. Nonetheless, these physical properties are an empirical measurement that cannot effectively describe the viscoelastic characteristics of bitumen and additionally fail to correlate well with asphalt mixture performance [29]. Likewise, Wu et al. [28] did not analyze the chemical variation of the functional groups of the bitumen, which is a useful indicator for evaluating the aging effect [30]. Therefore, a further analysis of rheological and chemical parameters is required for the evaluation of microwave heating on aging bitumen.

**Figure 1:**
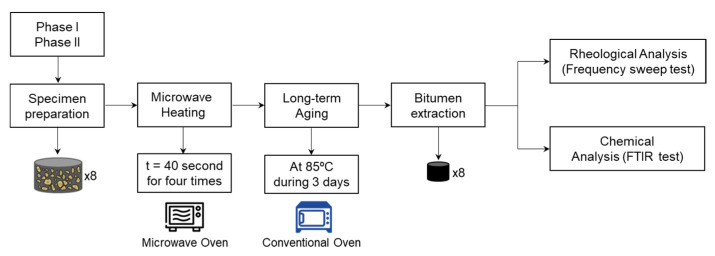
Schematic diagram of the experimental plan used in this research work.

Rheological characterization of bitumen is adopted in most studies about bitumen aging. However, chemical characterization is key to complement the rheological properties of bitumen, which provides the most effective method to confirm the aging oxidation of bitumen. The current research aims to evaluate the effect of microwave heating on the rheological and chemical properties of recovered bitumen. To achieve this objective, a comprehensive laboratory study was conducted using a conventional microwave oven to evaluate the microwave heating effects and a conventional heating oven to simulate the long-term aging. Rheological characterization was carried out with a Dynamic Shear Rheometer (DSR) by the values of the complex shear modulus (G*) and the phase angle (δ) based on the frequency sweep test. Fourier Transform Infrared Spectrometry (FTIR) tests were conducted to analyze the change in the molecular composition of the aged bitumen analyzing different bitumen samples exposed to microwave heating and long-term aging cycles. In brief, this study further details the mechanism of bitumen aging caused by the effects of microwave heating applied on asphalt mixture with crack self-healing purposes. The methodology followed to fulfill research objectives of this study is shown in Figure 1.

## 2. Materials and Methods

### 2.1. Materials and Manufacturing of Specimens

**Figure 2:**
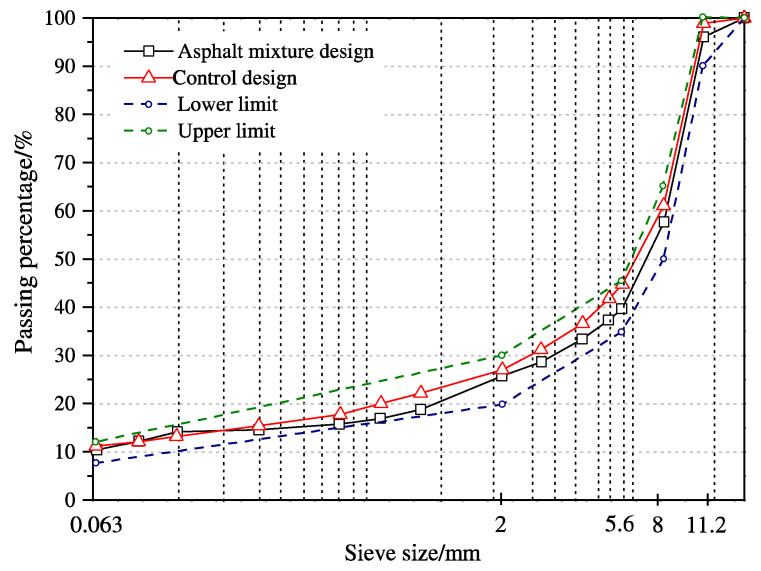
Sieving curve of the Stone Mastic Asphalt (SMA-11S) mixture used.

Stone mastic asphalt (SMA) with a maximum grain size of 11 mm (SMA 11S) was used. The SMA was composed of a strong coarse aggregate skeleton made of crushed diabase stones with a limestone mineral filler (grain size distribution shown in Figure 2). The used virgin bitumen was classified as 50/70 (1/10 mm) penetration grade, which is widely used in pavement engineering applications in Germany.

Several SMA test specimens were manufactured at a mixing temperature of 165 °C through two methods: (1) conventional SUPERPAVE preparation under laboratory conditions, and (2) preparation in asphalt plant. For the latter, SMA was industrially produced in a batch-type plant in Geilenkirchen (Germany). Afterward, SMA was transported to the test track of the institute of highway engineering at RWTH Aachen University (25 km distance) where it was laid down and compacted. Finally, several cores were drilled out from the track and delivered to TU Dresden for testing. The mixture’s bitumen content was 6.9% (per volume), the bulk density was 2436 kg/m^3^, and the void ratio was 2.1%.

### 2.2. Aging Modes of the Bitumen Samples

In this study, the bitumen material used was treated under two different aging modes:

- Mode I: bitumen used for the SMA manufacturing under method (1) was aged using hot air and UV light to simulate a long-term aging of 6 years of service life. In total, using this aged bitumen, eight SMA test specimens were prepared and tested.
- Mode II: bitumen within SMA cores only reproduced the short-term aging conditions during pavement construction. In total, eight SMA test specimens were evaluated under this mode.

In both modes, the aged bitumen was separated from the aggregates using a dissolution of trichloroethylene according to the standard EN12697-4 [31]. Furthermore, to contrast the rheological and chemical results of the samples exposed to microwave and long-term aging, the base bitumen was aged using the pressure aging vessel (PAV) method [32].

### 2.3. Microwave Heating and Long-Term Aging Cycles

**Figure 3:**
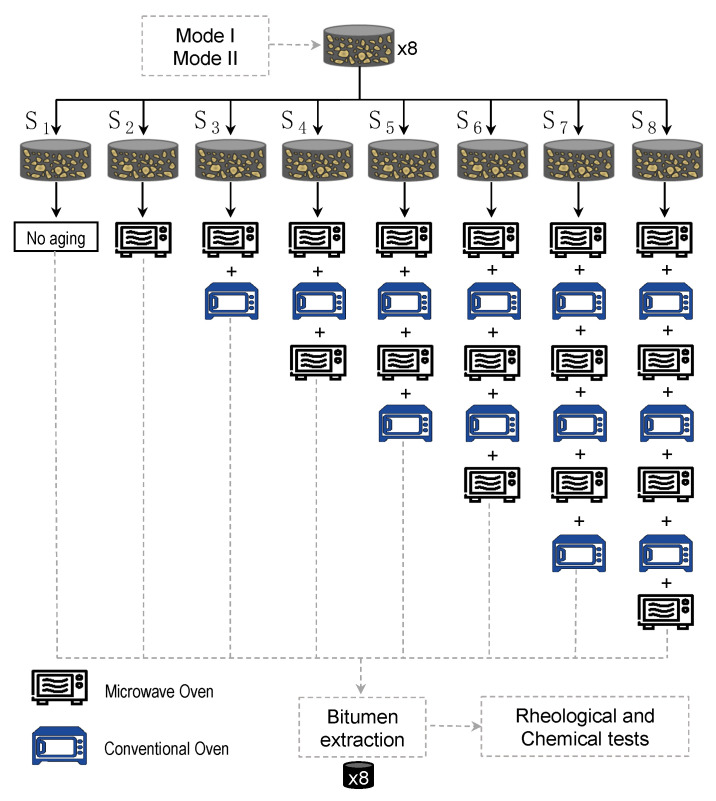
Test plan for microwave heating plus long-term aging cycle.

To study the aging effect on the bitumen under two different methods, the specimens were exposed to various cycles of microwave heating and long-term aging. The microwave heating was applied on the test specimens using a 900 W microwave oven with a working frequency of 2.45 GHz. The room temperature during the test was approximately 20 °C, and the initial temperature of the specimens was measured at five points of the surface with a laser thermometer, resulting in an average temperature of 22 °C. The test specimens were placed in the center of the microwave oven on an insulator material base and were heated for 40 s following the recommendations given by Norambuena-Contreras and Gonzalez-Torre [22], see test plan in Figure 3. The microwave heating was repeated four times with a 40 s rest period (i.e., without heating) to improve the heating distribution through the test specimens. The heating time was found suitable for microwave healing, because the measured surface temperature distribution was found similar to that obtained in previous research [1,23]. After microwave heating, the average surface temperature of the test specimens reached over 70 °C.

After aging by microwave heating, the long-term aging procedure was conducted according to the standard modified AASHTO R30 [33] for compacted asphalt mixtures. In this study, long-term aging was modified following the recommendations given by Elwardany et al. [34] in order to simulate a period of time related to the application of microwave heating in the field, which is expected to be applied every 3–5 years during maintenance activities. Hence, the test specimens were conditioned in a conventional oven at 85 °C ± 3 °C for 72 ± 0.5 h to simulate the long-term aging of a mixture in the field for a period over 3–5 years.

The test specimens were exposed to eight different stages of microwave heating plus long-term aging (S_1_–S_8_ in Figure 3) to compare the potential degree of aging that bitumen could have on its rheological and chemical properties. Figure 3 summarizes the experimental procedures for the microwave heating plus the long-term aging in conventional oven. In this figure, specimen S_1_ represents the control sample which was not exposed to any microwave heating or long-term aging cycle, whereas specimen S_8_ went through all cycles, i.e., it was exposed to four microwave heating and three long-term aging cycles. Once the experimental plan was completed, bitumen samples were recovered from the SMA test specimens to then perform the rheological and chemical tests.

### 2.4. Rheological Properties of Bitumen by DSR Tests

It is known that researchers have developed master curves to evaluated the influence of aging on the rheological properties of bitumen [35,36,37,38]. In this study, a dynamic shear rheometer (DSR) (Anton Paar MCR 502 Modular Compact Rheometer) was employed to perform frequency sweep tests at frequency ranging from 50 Hz to 0.5 Hz and at 10 different temperatures (−20, −10, 0, 10, 20, 30, 40, 50, 60, and 70 °C). The plates used in the DSR were 8 mm in diameter with a parallel plate geometry for low and intermediate temperatures in a range from −20 °C to 30 °C and 25 mm in diameter with a parallel geometry for higher temperatures in a range from 30 °C to 70 °C.

Dynamic oscillatory testing was performed under small strain-controlled conditions to ensure response within linear viscoelastic range (LVE). The LVE range was denoted by the strain value in which the dynamic shear modulus equates to 95% of the initial value [39]. The time–temperature superposition principle (TTPS) is used to construct master curves from LVE data by shifting measurement at different temperatures in order to obtain a continuous curve at the reference temperature [40].

On the basis of the time–temperature superposition principle and frequency sweep test results, G* and δ master curves were constructed. To create the master curve, an arbitrary reference temperature of 30 °C was used, and the data collected from frequency sweeps at all other temperatures were shifted to the reference temperature by shift factors. The shift factors were calculated using a Williams–Landel–Ferry (WLF) equation [41] as follows:

(1) $$\;\log\alpha \left(T\right)=-\frac{C_{1}\left(T-T_{R}\right)}{C_{2}+\left(T-T_{R}\right)},$$

where α(T) is the shifting factor relative to the reference temperature, T is the initial temperature (°C), $T_{R}$ is the arbitrarily chosen reference temperature (°C), and $C_{1}$, $C_{2}$ are fitting constants.

Master curves for G* and δ were plotted as a function of reduced frequency $\left(f_{r}\right)$ at the defining temperature on a log_30_-log and semi-log scale, respectively. The $f_{r}$ is a function of the shift factor logα(T) and the frequency (f) and is calculated using the following equation [42]:

(2) $$\;f_{r}=f\cdot 10^{\log\alpha \left(T\right)}.$$

### 2.5. Quantification of the Rheological Aging Indexes

A common methodology for assessing the aging performance of bitumen is primarily through the measurement of specific parameters before and after aging. These parameters are normally related to the physical, chemical, and rheological properties [38]. In the present study, the changes in the rheological properties after microwave heating and long-term aging were evaluated by rheological aging indices. The rheological aging indices adopted for this research were obtained from the measurement of rheological properties in the frequency sweep test, which were the complex modulus index (${\mathrm{AI}}_{G^{*}}$) and phase angle index (${\mathrm{AI}}_{\delta }$). The following equations show the aging indices used in this research:

(3) $$AI_{G^{*}}=\frac{G_{Aged}^{*}}{G_{Unaged}^{*}},$$

(4) $$\;AI_{\delta }=\frac{\delta_{Aged}}{\delta_{Unaged}},$$

where $G_{Unaged}^{*}$, $\delta_{Unaged}$ are the complex modulus and phase angle of the unaged bitumen, which are represented by the control bitumen sample, i.e., *bitumen 1*, and $G_{Aged}^{*}$, $\delta_{Aged}$ are the complex modulus and phase angle of the aged recovered bitumen samples, which were exposed to different microwave heating and long-term aging cycles (see Figure 3).

### 2.6. Chemical Properties of Bitumen by FTIR Tests

As previously mentioned, during the oxidation process, chemical variations that occur refer to the formation of carbonyl groups (C=O) and sulfoxide groups (S=O), which increase the overall polarity of the bitumen [6], affecting its physical and rheological properties [5]. Fourier-transform infrared spectroscopy (FTIR) is a successful experimental technique to analyze the changes in the chemical composition of bitumen due to oxidative aging [43].

A Nicolet iS5 FTIR spectrometer was used in this study to identify the chemical functional groups of the recovered bitumen after the microwave heating and long-term aging cycles. Each spectrum was scanned 100 times at a resolution of 4 cm^−1^ and recorded in a wavenumber range from 4000 to 600 cm^−1^. The changes caused by aging can be found between 2000 and 600 cm^−1^. These wavenumbers correspond to functional groups related to the oxidation process [44]. The peaks of the carbonyl and sulfoxide groups can be found at wavenumbers 1700 and 1030 cm^−1^, respectively [44].

To evaluate the aging of bitumen, both a carbonyl index (I_C=O_) and a sulfoxide index (I_S=O_) were determined [38]. The indices of these groups can be calculated from the peak area of their bands and divided by the sum of all bands in wavenumbers ranging from 2000–600 cm^−1^ [45]. The relative ratios of the areas of C=O and S=O were calculated using the following equations [37,43,46]:

(5) $$I_{C=O}=\frac{A_{1700}}{\sum A},$$

(6) $${\;I}_{S=O}=\frac{A_{1030}}{\sum A},$$

(7) $$\sum A=A_{1700}+A_{1600}+A_{1460}+A_{1376}+A_{1030}+A_{864}+A_{814}+A_{743}+A_{724},$$

where $A_{1030}$ represents the area of sulfoxide peaks, $A_{1700}$ represents the area of carbonyl peaks, and ∑A is the sum of areas of all bands in wavenumber range of 2000–600 cm^−1^. The peak areas were evaluated using numerical integration provided by OriginPro software.

### 2.7. Summary Description of the Tested Bitumen Samples

Table 1 summarizes the different bitumen samples experimentally analyzed in this study. In addition to the eight bitumen samples, a fresh or virgin bitumen sample and an aged bitumen sample were analyzed for comparison purposes. The bitumen was aged applying the pressure aging vessel (PAV) method.

## 3. Results and Discussion

### 3.1. Effect of the Microwave Heating and Long-Term Aging on Bitumen from Mode I

#### 3.1.1. Effect on the Rheological Properties of Bitumen Samples from Mode I

**Figure 4:**
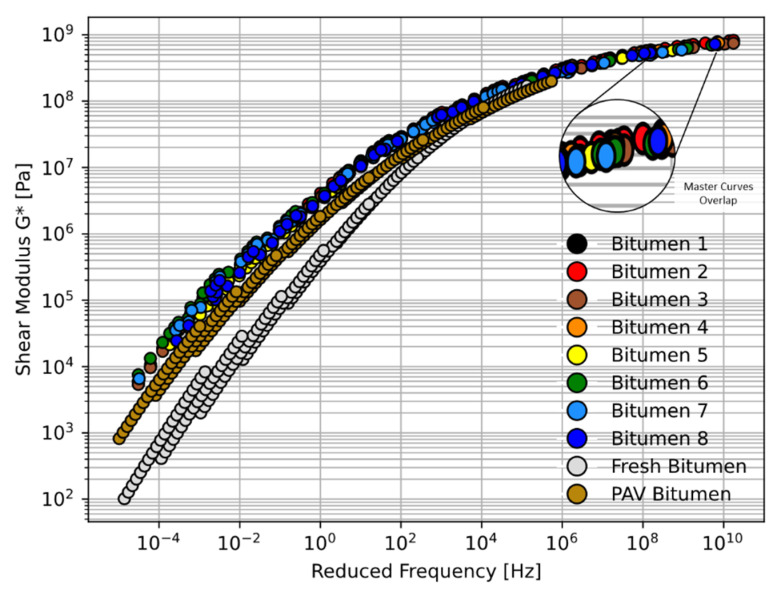
Master curve comparison for all bitumen samples from Mode I in terms of G*.

**Figure 5:**
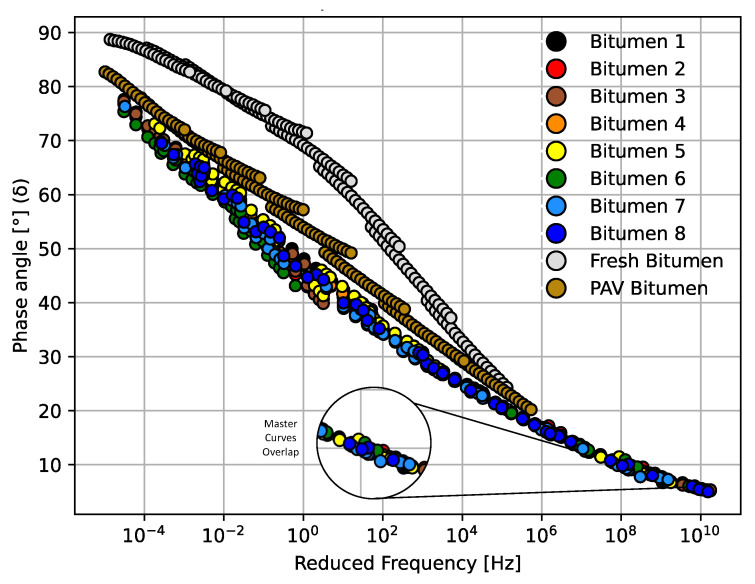
Master curve comparison for all bitumen samples from Mode I in terms of δ.

Figure 4 and Figure 5 summarize all the results of rheological properties measured for the bitumen samples from Mode I (see Section 2.2) exposed to different cycles of microwave heating and long-term aging. The results of the viscoelastic parameters complex modulus (G*) and phase angle (δ) are presented in master curves at a reference temperature of 30 °C. Both master curves for G* and δ were plotted as a function of reduced frequency. In these figures, the aging of the bitumen samples can be observed in master curves when G* increases and δ decreases [37].

As can be seen in Figure 4 and Figure 5, all the master curves for bitumen 1–8 overlap. Hence, no obvious differences can be seen in the variation of G* through the different microwave heating and long-term aging cycles. Figure 5 shows an unexpected result for bitumen 6, with a few degrees lower than bitumen 8. This result was attributed to testing variability. The overlap tendency shown in Figure 4 and Figure 5 can be attributed to the fact that, once the bitumen or asphalt is aged, the additional aging due to microwave heating has no significant effect on the aging performance of G* and δ. From low frequency to high frequency, the spacing for the G* master curve of all bitumen samples was minor. However, at higher frequency, all samples tended to reach an asymptote at value of 10^6^ Pa. Figure 5 shows the master curves for the phase angle with an overlap trend between the eight bitumen specimens. No clear effect can be seen in the phase angle through the various microwave heating and long-term aging cycles.

Additionally, Figure 4 and Figure 5 show the G* and δ master curves for a fresh and a PAV bitumen. From these figures, it can be observed that the rheological properties at low frequency show that fresh and PAV bitumen had lower values of G* and higher values of δ, which means that fresh and PAV bitumen samples were less aged than the other bitumen samples exposed to the microwave and long-term aging cycles. This is an interesting phenomenon to be noted considering that bitumen samples 1 to 8 were initially exposed to long-term aging (conventional oven at 85 °C ± 3 °C for 72 ± 0.5 h). It would be expected that this method of long-term aging promotes the oxidation process, increasing G* and decreasing δ gradually with the cycle increase; however, this result cannot be seen in the master curves because the bitumen was very aged. It should be noted that this result is consistent, because the oxidation process for old bitumen increases at a very low rate according to the literature [47].

**Figure 6:**
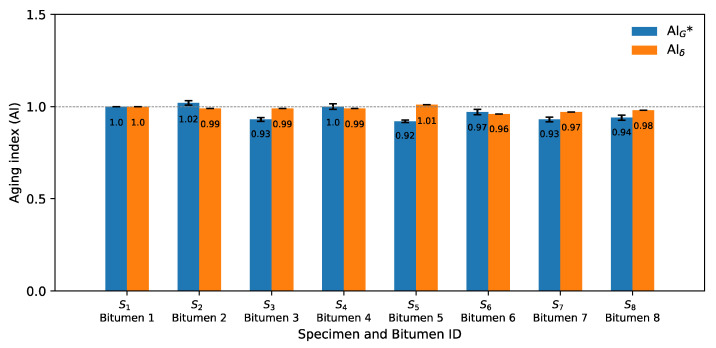
Aging indices using G* and δ for criterion 1 (20 °C, 10 Hz) from Mode I.

**Figure 7:**
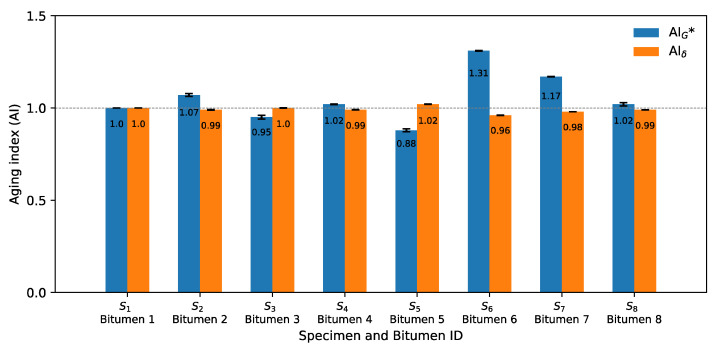
Aging indices using G* and δ for criterion 2 (60 °C, 1 Hz) from Mode I.

Hence, to quantify the effect of the microwave and long-term aging on the rheological properties of bitumen from Mode I, the rheological aging indices (AI) were calculated using two criteria. The first was to calculate the AI at 20 °C and frequency 10 Hz, representing the typical design considerations, and the second was to calculate the AI at 60 °C and frequency 1 Hz, established as the high-temperature and low-frequency condition that corresponds closely to permanent deformation conditions. Thus, Figure 6 and Figure 7 present the average results of three replicates (± one standard deviation error bar) of the rheological aging indices for the eight bitumen samples at two different criteria, respectively. Average results were calculated using three values. Figure 6 shows the aging index of G* and δ for the first criterion. The *x*-axis shows the identification of the specimen, along with the number of its associated recovered bitumen. For example, for bitumen 1 (which is related to S_1_), it is indicated that there was no microwave heating and no long-term aging cycle (control sample); therefore, it had a value of $AI_{G^{*}}$ and $AI_{\delta }$ of 1.0.

In Figure 6, no clear effect is observed in the $AI_{G^{*}}$ because some bitumen samples increased and others decreased the aging index value as the microwave heating increased and the long-term aging cycles were extended. The increase in $AI_{G^{*}}$ was expected to be gradual, due to the increase in the complex modulus by the oxidation process with the long-term aging procedure. The trend in the $AI_{\delta }$ was also variable as microwave cycles and long-term aging increased. There was not a gradual trend as expected. The most important variation in $AI_{G^{*}}$ can be seen in bitumen 2, where, after one microwave heating cycle (S_2_), the G* increased by 2% (*p* < 0.001, calculated with Student’s *t*-test and 95% confidence level) in relation to bitumen 1. In the case of $AI_{\delta }$, the most important variation was in bitumen 6, which, after three microwave heating and two long-term aging cycles (S_6_), the phase angle decreased by 4% (*p* < 0.001) in relation to bitumen 1. This result confirms that the effect on the aging performance rheological properties of bitumen samples (G*, δ) was minor.

Furthermore, Figure 7 shows the aging index of G* and δ for the second criterion. A similar trend to that of $AI_{G^{*}}$ and $AI_{\delta }$ at 20 °C (see Figure 6) can be seen at 60 °C. A variable behavior can be noted in the indices and not a gradual tendency as expected, since, as microwave heating and long-term aging cycles increased, the oxidation process also increased, generating a gradual increase in $AI_{G^{*}}$ and a gradual decrease in $AI_{\delta }$. An unexpected result of $AI_{G^{*}}$ can be noted in bitumen 6 and 7, related to the control bitumen, where $AI_{G^{*}}$ increased by 31% (*p* < 0.001) and 17% (*p* < 0.001).

#### 3.1.2. Effect on the Chemical Properties of Bitumen Samples from Mode I

**Figure 8:**
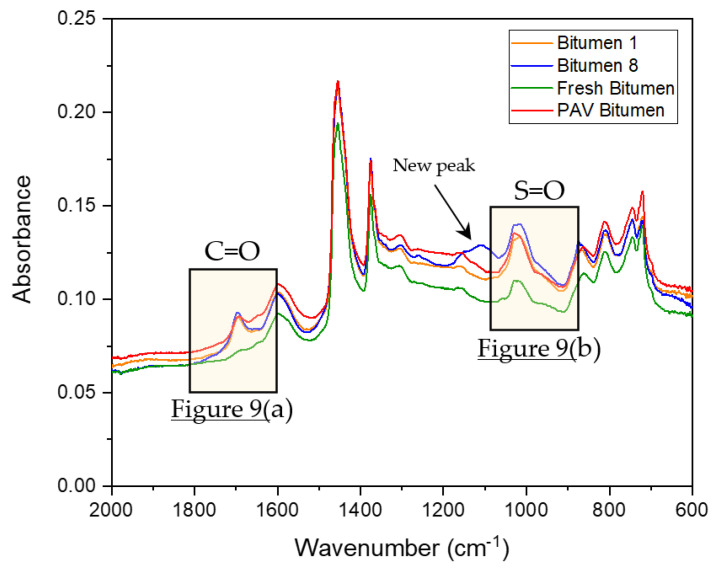
FTIR spectra of bitumen samples from Mode I in the range 2000–600 cm^−1^.

**Figure 9:**
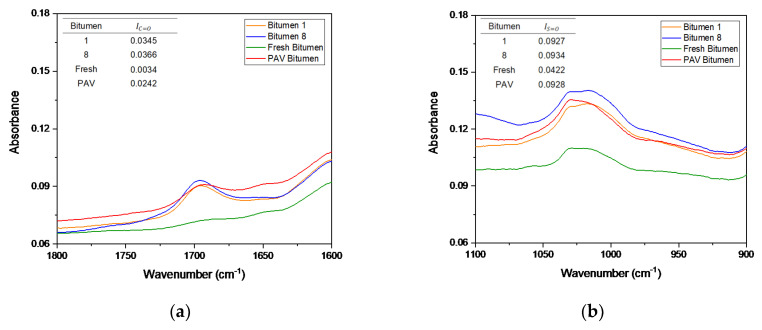
FTIR spectra of (**a**) C=O carbonyl and (**b**) S=O sulfoxide peaks of bitumen samples from Mode I.

On the basis of DSR results, only bitumen samples 1 and 8 were selected to compare the chemical changes when the bitumen is exposed to microwave heating and long-term aging cycles. Additionally, fresh bitumen 50/70 pen (without any artificial aging treatment) and PAV-aged bitumen were analyzed to contrast with bitumen 1 and 8 in terms of FTIR. The FTIR spectral results ranging from 2000–600 cm^−1^ wavenumbers, covering the regions with the main oxidative aging products [48], are shown in Figure 8. In Figure 8, the absorption bands of the C=O and S=O groups of the bitumen were in a wavenumber range centered around 1700 cm^−1^ and 1030 cm^−1^, respectively. The trend for all bitumen samples was almost the same. However, as shown by the arrow in Figure 8, the FTIR spectrum of bitumen 8 (blue line), there were new absorption peaks around approximately 1100 cm^−1^. These results indicate that a new functional group was generated, which can be attributed to the molecular interactions and chemical composition changes of the bitumen under microwave heating and long-term aging cycles. To appreciate the formation of an oxidation product, Figure 9 shows a closer view of the carbonyl and sulfoxide peaks framed in Figure 8.

In Figure 9a, the absorption peaks for the carbonyl group can be seen, where bitumen samples 1 (S_1_) and 8 (S_8_) show a similar trend. However, the absorption of bitumen 8 was slightly higher than that of bitumen 1. In addition, at the peak 1700 cm^−1^, the PAV bitumen showed a similar absorption to bitumen 1, but lower absorption than bitumen 8. Moreover, the area amplitude for PAV bitumen was less than that for bitumen 1 and 8. Fresh bitumen had no peaks of the carbonyl group. Likewise, in Figure 9b, the sulfoxide group can be observed in the FTIR spectra, occurring due to thermo-oxidative aging during the production and storage of bitumen [44]. Figure 9b demonstrates that bitumen 1 and the PAV bitumen had a similar tendency in the sulfoxide zone. Additionally, bitumen 8 had a higher absorption in the sulfoxide zone than all bitumen samples. The obtained spectral carbonyl and sulfoxide indices were calculated using Equations (6) and (7) to quantify the aging degree. The tables in Figure 9a,b present the carbonyl index ($I_{C=O}$) and sulfoxide index ($I_{S=O}$) of the different bitumen samples analyzed, respectively. The tables show that the carbonyl and sulfoxide indices increased as the microwave heating and long-term aging cycles increased. Moreover, it should be noted that, after four microwave heating and three long-term aging cycles, bitumen 8 (S_8_) slightly increased the $I_{C=O}$ and $I_{S=O}$ compared with bitumen 1 (S_1_). Furthermore, fresh bitumen had the lowest rates, which is consistent with its virgin bitumen condition. PAV bitumen had a lower carbonyl index compared to bitumen 1 and 8, a similar sulfoxide index to bitumen 1, and a lower sulfoxide index than bitumen 8. These results are consistent with the literature because (1) the carbonyl functional group is related to the increase in viscosity by effect of the aging of bitumen, and (2) the sulfoxide functional group is usually produced in higher amounts than the carbonyl group [43]. Thus, the variation of carbonyl and sulfoxide groups represents the oxidation degree and further reflects the aging degree of bitumen. In short, the difference between the carbonyl index values for bitumen 1 (control sample) and bitumen 8 was smaller (0.0021), demonstrating the minor effects of aging on bitumen due to microwave heating and long-term aging cycles.

### 3.2. Effect of the Microwave Heating and Long-Term Aging on Bitumen from Mode II

#### 3.2.1. Effect on the Rheological Properties of Bitumen Samples from Mode II

**Figure 10:**
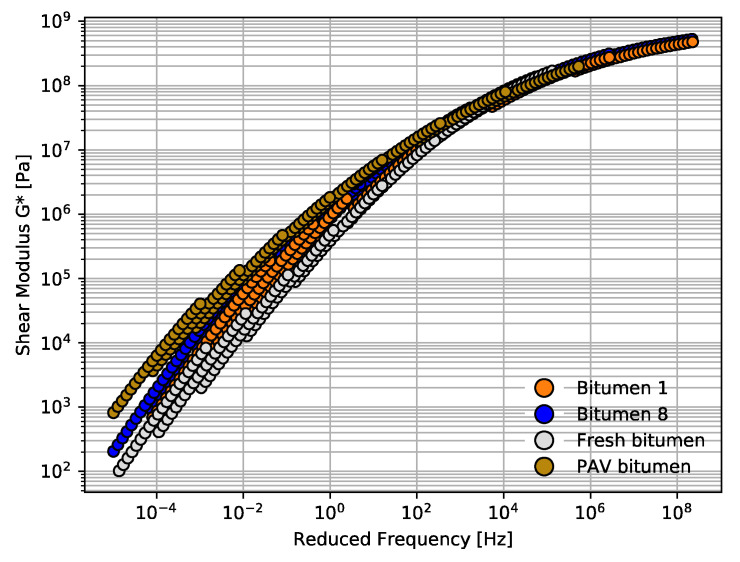
Master curve comparison for bitumen samples 1 and 8 from Mode II in terms of G*.

**Figure 11:**
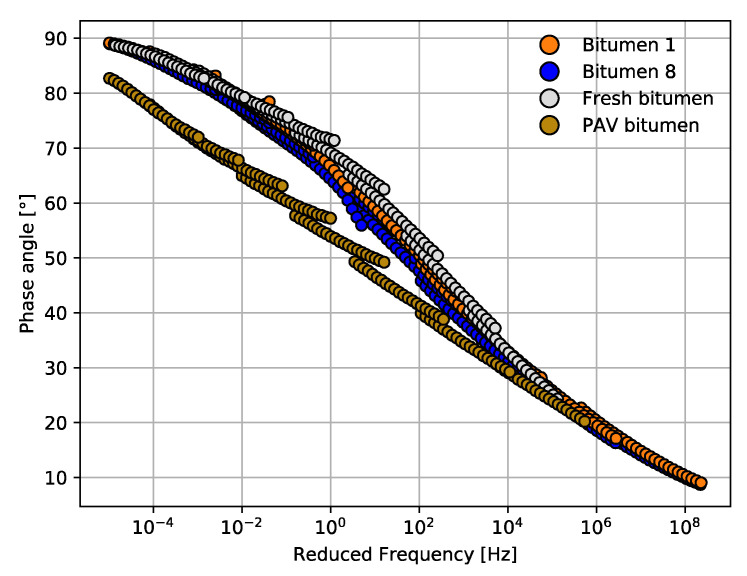
Master curve comparison for bitumen samples 1 and 8 from Mode II in terms of δ.

In Mode II, a not-so-aged asphalt mixture was compared with Mode I. Thus, in this mode and analogously to Mode I (discussed in previous section), a comparison of master curves across bitumen 1 and 8 was carried out with the aim of analyzing the specimens most and least exposed to the microwave and long-term aging cycles. The master curves of G* and δ at a reference temperature of 30 °C are presented in Figure 10 and Figure 11, respectively. As shown in Figure 10, the difference in the G* master curve for bitumen 8 was narrow compared to that of bitumen 1. There were slight differences after four microwave heating and three long-term aging cycles (S_8_) for bitumen 8; the G* range of bitumen 8 was about 205 to 5.30 × 10^8^ Pa and that of the bitumen 1 was 602 to 4.98 × 10^8^ Pa. Furthermore, Figure 11 shows that the δ master curve for bitumen 8 was slightly lower than that for bitumen 1, which indicates that bitumen 8 was more aged by the effect of the heating aging cycles.

Furthermore, fresh and PAV master curves were also drawn in Figure 10 and Figure 11 to compare the aging level of the recovered bitumen samples. In Figure 10, PAV bitumen can be seen as the most aged bitumen, since the master curves present greater values of G*. In contrast, in Figure 10, fresh bitumen shows a master curve with lower values of G*. Although bitumen 8 was exposed to four microwave heating and three long-term aging cycles (S_8_), the master curve was lower than that of PAV bitumen, which shows that the aging cycles may not present as much damage due to aging as expected. This conclusion coincides with the observed rheological behavior for the bitumen samples tested in Mode I.

**Figure 12:**
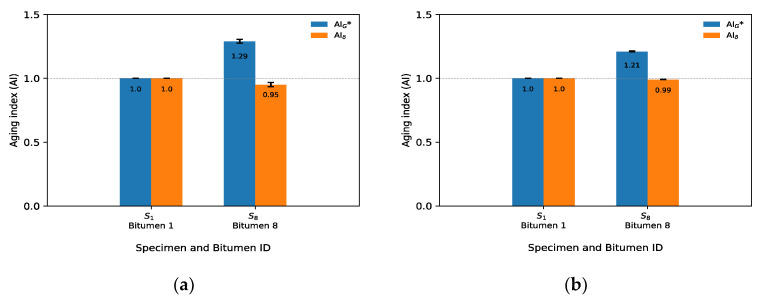
Aging indices of Mode II for two criteria: (**a**) 20 °C, 10 Hz; (**b**) 60 °C, 1 Hz.

Similarly, in Figure 11, at lower and intermediate frequency, there was an important difference in the phase angle master curves. PAV had the lower values of δ, which indicates that PAV bitumen had the most important aging in comparison with all bitumen samples. Hence, to quantify the effect of the microwave heating and long-term aging on the rheological properties of bitumen samples 1 and 8 from Mode II, the rheological aging indices (*AI*) were calculated according to the same criteria as for Mode I, i.e., at 20 °C, 10 Hz and 60 °C, 1 Hz, as shown in Figure 12.

From Figure 12, it can be observed that, as microwave heating and long-term aging cycles increased, $AI_{G^{*}}$ increased and $AI_{\delta }$ decreased. This result is caused by the oxidation process of the chemical components of the bitumen, which impacts the mechanical properties of the bitumen, with the samples becoming more solid-like, as indicated by the increased G* and decreased δ. If the aging indices at 20 °C and 10 Hz are compared in Figure 12a, bitumen 8 increased by 29% (*p* < 0.001, calculated with Student’s *t*- test and 95% confidence level) in relation to bitumen 1. In Figure 12b, the aging index for bitumen 8 increased by 21% (*p* < 0.001) in relation to bitumen 1. For $AI_{\delta }$, at 20 °C and 10 Hz, bitumen 8 decreased the aging index by 5% (*p* = 0.0015), and, at 60 °C and 1 Hz, the variation of the $AI_{\delta }$ was 1% (*p* < 0.001), both in relation to bitumen 1.

#### 3.2.2. Effect on the Chemical Properties of Bitumen Samples from Mode II

**Figure 13:**
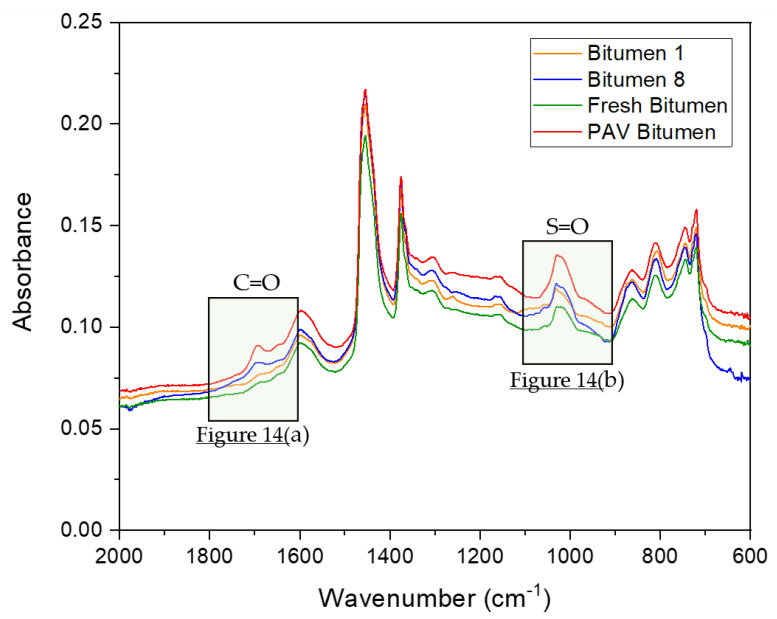
FTIR spectra of bitumen samples from Mode II in the range 2000–600 cm^−1^.

In Mode II, the same chemical analyses were conducted for bitumen 1 and 8 to analyze the chemical composition changes of bitumen at different aging levels by effect of the microwave heating and long-term aging. Thus, fresh bitumen 50/70 pen and PAV-aged bitumen were analyzed to contrast with bitumen 1 and 8 in terms of FTIR. The spectra collected from 2000–600 cm^−1^ are shown in Figure 13. Figure 13 shows that (1) the trend for bitumen 1 and 8 were almost the same and, thus, no new functional groups were generated during the oxidation process with either the microwave and long-term aging cycles, and (2) with the increase in the aging severity, the absorption spectra gradually increased. According to Figure 13, peaks at 1030 cm^−1^ attributed to the stretch vibration of the sulfoxide group were observed in all bitumen samples. However, peaks at 1700 cm^−1^ related to the carbonyl group were only observed in the FTIR spectra of PAV and bitumen 8.

**Figure 14:**
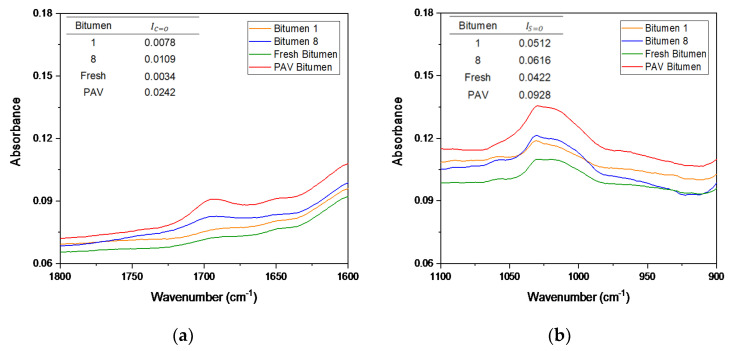
FTIR spectra of (**a**) C=O carbonyl and (**b**) S=O sulfoxide peaks of bitumen samples from Mode II.

For a better appreciation of the formation of the oxidation product, Figure 14 shows a closer view of the carbonyl and sulfoxide peaks framed in Figure 13. From Figure 14a, it can be observed that the PAV bitumen presented greater absorption in the carbonyl zone than the other bitumen samples. Moreover, no carbonyl group peaks were observed for bitumen 1 and fresh bitumen. In the carbonyl zone (see Figure 14a), bitumen 8 had a slighter increase in absorption compared to bitumen 1. In contrast, Figure 14b shows that the peaks of the sulfoxide group were similar for all bitumen samples, while the ratio of the peak area was different. The amplitude for the area increased as the aging level of the bitumen increased. In particular, the amplitude for the area of PAV bitumen was the greatest. In addition to the spectral observation, the effect of aging was evaluated by the carbonyl and sulfoxide indices. The tables in Figure 14a,b present the carbonyl index ($I_{C=O}$) and sulfoxide index ($I_{S=O}$) of the different bitumen samples analyzed from Mode II, respectively.

After four microwave heating and three long-term aging cycles, the $I_{C=O}$ for bitumen 8 reached 0.0109, which is 1.4 times higher than the $I_{C=O}$ for bitumen 1. Comparing the results with PAV bitumen samples, it can be seen that $I_{C=O}$ reached 0.0244, which demonstrated that bitumen aging had a much higher effect than in bitumen 1 and 8. In the case of $I_{S=O}$, bitumen 8 measured 1.2 times higher than bitumen 1, and its value gradually increased as the bitumen aging severity increased, with the PAV bitumen having the greatest index, showing the minor effects of aging on bitumen by effect of microwave heating and long-term aging cycles. This result is consistent with the obtained conclusions for Mode I.

### 3.3. Relationship between Chemical and Rheological Results from Mode I and II

**Figure 15:**
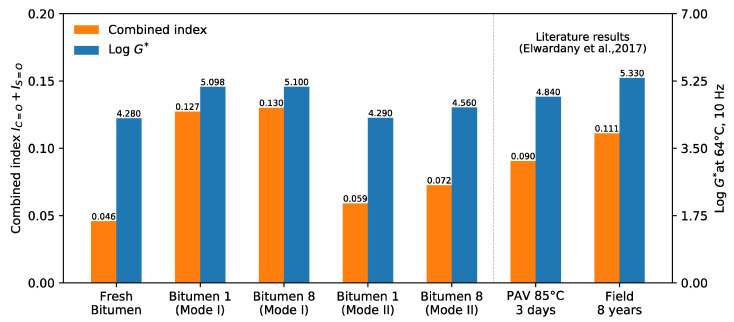
Relationship between chemical and rheological results from Mode I and II.

It is widely recognized that the performance of an asphalt mixture is largely dependent on the rheological behavior of bitumen [49]. At the same time, the rheological properties depend on the chemical changes in the bitumen [7]. Qin et al. [50] found a linear relationship between rheological parameters and chemical composition, such as the FTIR absorbance given by the sum of carbonyl and sulfoxide indices. In this context, Elwardany et al. [34] and Ge et al. [30] confirmed this behavior, where G* increased consistently when the sum of the carbonyl index ($I_{C=O}$) and sulfoxide index ($I_{S=O}$) increased. Figure 15 presents the relationship between the sum of the carbonyl and sulfoxide indices ($I_{C=O\;}+I_{S=O}$) and log G* at 64 °C and 10 Hz from the obtained results for Mode I and II and the literature results [34]. As can be seen, as the sum of $I_{C=O\;}$ and $I_{S=O}$, increased, the log of the complex modulus at 64 °C and 10 Hz also increased.

Additionally, Figure 15 shows two literature results corresponding to bitumen recovered from a compacted specimen exposed during a procedure of PAV (3 days at 85 °C and 300 kPa air pressure) and another from an 8 year old field core [34], in order to contrast the aging level of the samples in Mode I and II. As can be seen, the results of Mode I for bitumen 1 and 8 were very similar to the results of the 8-year-old field core. On the other hand, the results of log G* at 64 °C for Mode II were lower than for the PAV bitumen, which simulated a long-term aging of approximately 5 years [34]. Comparing all the results of Mode I and II, it can be concluded that the changes in chemical and rheological properties were very small, mainly resulting from the viscoelastic properties that changed at a lower rate for aged materials [47]. The opposite can be seen in Mode II, where the material used was newer; after different microwave heating and long-term aging cycles, the changes were greater.

From a chemical perspective, the hydrocarbon reaction of the free radical of the bitumen with oxygen is responsible for most of the oxidative aging [51]. Aging can be summarized as a process in which the chemical components of the bitumen vary, are consumed, and increase. The components move from more nonpolar fractions to the more polar fractions as oxygen-containing functional groups are formed in the asphalt [52]. As the time of aging increases, the free radical production begins to decrease. Initially, there is a rapid reaction followed by a slower, constant reaction [51]. This may be an explanation for the results obtained in Mode I, where the oxidation process did not have a clear behavior. Comparing bitumen 1 and 8 proved that the presence of aging, as reflected in the chemical and rheological properties, was low.

## 4. Conclusions

This paper evaluated the effect of microwave heating and long-term aging on recovered bitumen through rheological and chemical properties. On the basis of the test results, the following conclusions were drawn:

- Microwave heating and long-term aging have no significant effect on the aging performance of G* and δ for aged asphalt mixtures. However, the rheological properties of bitumen showed minor aging effects with microwave heating and long-term aging cycles for newer asphalt mixtures.
- It was expected that modified AASHTO R30 would promote the oxidation process, increasing G* and decreasing δ gradually; however, the long-term standard did not oxidize the samples as expected. The study of microwave aging together with aging by modified AASHTO R30 did not permit to clearly differentiate the aging due to microwave heating and the AASHTO R30 accelerated procedure.
- According to the FTIR results, as the microwave heating and long-term aging cycles increased, the carbonyl and sulfoxide indices increased in both phases. Therefore, bitumen aging influences chemical changes in bitumen, including the formation of carbonyl and sulfoxide compounds.
- It was possible to confirm that changes in the molecular composition of the samples varied the viscoelastic properties of the bitumen, as shown in DSR tests.
- A strong relationship could be observed between the chemical and rheological results, showing that both properties are good indicators to evaluate bitumen aging.
- Overall, this study confirmed that microwave heating is a valuable alternative for the maintenance of asphalt pavements, without severely affecting the rheological and chemical properties of bitumen.

## Figures and Tables

**Table 1:** Symbology of the bitumen samples tested in this study.

Symbology	Description
Bitumen 1	Bitumen sample recovered from SMA without stages of aging ^1^
Bitumen 2–8	Bitumen samples recovered from SMA test specimens exposed to different microwave and oven stages of aging ^1^
Fresh Bitumen	Virgin bitumen sample without any treatment
PAV Bitumen	Bitumen sample aged using the pressure aging vessel (PAV) method

^1^ See Figure 3.

## Data Availability

The data presented in this study are available on request from corresponding author.

## References

[1] González A., Norambuena-Contreras J., Storey L., Schlangen E. (2018). Effect of RAP and fibers addition on asphalt mixtures with self-healing properties gained by microwave radiation heating. Constr. Build. Mater..

[2] Kim Y., Little D.N., Asce F., Lytton R.L., Asce F.P.E. (2003). Fatigue and Healing Characterization of Asphalt Mixtures. J. Mater. Civil Eng..

[3] Rochlani M., Leischner S., Falla G.C., Wang D., Caro S., Wellner F. (2019). Influence of filler properties on the rheological, cryogenic, fatigue and rutting performance of mastics. Constr. Build. Mater..

[4] Tauste R., Moreno-Navarro F., Sol-Sánchez M., Rubio-Gámez M.C. (2018). Understanding the bitumen ageing phenomenon: A review. Constr. Build. Mater..

[5] Sirin O., Paul D.K., Kassem E. (2018). State of the Art Study on Aging of Asphalt Mixtures and Use of Antioxidant Additives. Adv. Civ. Eng..

[6] Hunter R.N., Self A., Read J. (2015). The Shell Bitumen Handbook.

[7] Miró R., Martínez A.H., Moreno-Navarro F., del Carmen Rubio-Gámez M. (2015). Effect of ageing and temperature on the fatigue behaviour of bitumens. Mater. Des..

[8] Sandoval G., Thenoux G., Molenaar A.A.A., Gonzalez M. (2016). The antioxidant effect of grape pomace in asphalt binder. Int. J. Pavement Eng..

[9] Gallego J., Del Val M.A., Contreras V., Páez A. (2013). Heating asphalt mixtures with microwaves to promote self-healing. Constr. Build. Mater..

[10] Miao P., Wang S., Liu W. (2017). Improving microwave absorption efficiency of asphalt mixture by enriching Fe_3_O_4_ on the surface of steel slag particles. Mater. Struct. Constr..

[11] Norambuena-Contreras J., Serpell R., Valdés Vidal G., González A., Schlangen E. (2016). Effect of fibres addition on the physical and mechanical properties of asphalt mixtures with crack-healing purposes by microwave radiation. Constr. Build. Mater..

[12] González A., Valderrama J., Norambuena-Contreras J. (2019). Microwave crack healing on conventional and modified asphalt mixtures with different additives: An experimental approach. Road Mater. Pavement Des..

[13] Ayar P., Moreno-Navarro F., Rubio-Gámez M.C. (2016). The healing capability of asphalt pavements: A state of the art review. J. Clean. Prod..

[14] Xu S., Liu X., Tabaković A., Schlangen E. (2021). The prospect of microwave heating: Towards a faster and deeper crack healing in asphalt pavement. Processes.

[15] Nalbandian K.M., González Á. (2021). Assessment of self-healing asphalt pavement fatigue life using analytical Jc approach and laboratory results. Constr. Build. Mater..

[16] González A., Norambuena-Contreras J., Storey L., Schlangen E. (2018). Self-healing properties of recycled asphalt mixtures containing metal waste: An approach through microwave radiation heating. J. Environ. Manag..

[17] Gómez-Meijide B., Ajam H., Lastra-González P., Garcia A. (2018). Effect of ageing and RAP content on the induction healing properties of asphalt mixtures. Constr. Build. Mater..

[18] Norambuena-Contreras J., Gonzalez A., Concha J.L., Gonzalez-Torre I., Schlangen E. (2018). Effect of metallic waste addition on the electrical, thermophysical and microwave crack-healing properties of asphalt mixtures. Constr. Build. Mater..

[19] García A., Schlangen E., Van De Ven M., Van Vliet D. (2010). Induction heating of mastic containing conductive fibers and fillers. Mater. Struct..

[20] García A., Norambuena-Contreras J., Partl M.N. (2013). A parametric study on the influence of steel wool fibers in dense asphalt concrete. Mater. Struct..

[21] Liu Q., Schlangen E., Van De Ven M., Van Bochove G., Van Montfort J. (2012). Evaluation of the induction healing effect of porous asphalt concrete through four point bending fatigue test. Constr. Build. Mater..

[22] Norambuena-Contreras J., Gonzalez-Torre I. (2017). Influence of the microwave heating time on the self-healing properties of asphalt mixtures. Appl. Sci..

[23] Norambuena-Contreras J., Garcia A. (2016). Self-healing of asphalt mixture by microwave and induction heating. Mater. Des..

[24] Flores G., Gallego J., Giuliani F., Autelitano F. (2018). Aging of asphalt binder in hot pavement rehabilitation. Constr. Build. Mater..

[25] Tang J., Liu Q., Wu S., Ye Q., Sun Y., Schlangen E. (2016). Investigation of the optimal self-healing temperatures and healing time of asphalt binders. Constr. Build. Mater..

[26] Amani S., Kavussi A., Karimi M.M. (2020). Effects of aging level on induced heating-healing properties of asphalt mixes. Constr. Build. Mater..

[27] Yalcin E. (2021). Effects of microwave and induction heating on the mechanical and self-healing characteristics of the asphalt mixtures containing waste metal. Constr. Build. Mater..

[28] Wu S., Yang J., Yang R., Zhu J., Liu S. (2018). Investigation on Microwave Heating Technology for Rutting Maintenance in Asphalt Pavement. J. Test. Eval..

[29] Airey G.D. (2002). Use of Black Diagrams to Identify Inconsistencies in Rheological Data Use of Black Diagrams to Identify Inconsistencies in Rheological Data. Road Mater. Pavement Des..

[30] Ge D., Chen S., You Z., Yang X., Yao H., Ye M., Yap Y.K. (2019). Correlation of DSR Results and FTIR’s Carbonyl and Sulfoxide Indexes: Effect of Aging Temperature on Asphalt Rheology. J. Mater. Civ. Eng..

[31] European Commitee for Standarization (2013). EN 12697-4: Bituminous Mixtures.Test Methods for Hot Mix Asphalt. Bitumen Recovery: Fractionating Column.

[32] European Commitee for Standarization (2012). Bitumen and Bituminous Binders—Accelerated Long-Term Ageing Conditioning by a Pressure Ageing Vessel (PAV) (EN 14769).

[33] American Association of State Highway and Transportation (AASHTO) (2002). Standard Practice for Mixture Conditioning of Hot-Mix Asphalt (HMA) (AASHTO R30).

[34] Elwardany M.D., Yousefi Rad F., Castorena C., Kim Y.R. (2017). Evaluation of asphalt mixture laboratory long-term ageing methods for performance testing and prediction. Road Mater. Pavement Des..

[35] Zapién-Castillo S., Rivera-Armenta J.L., Chávez-Cinco M.Y., Salazar-Cruz B.A., Mendoza-Martínez A.M. (2016). Physical and rheological properties of asphalt modified with SEBS/montmorillonite nanocomposite. Constr. Build. Mater..

[36] Gao J., Wang H., You Z., Rosli M., Hasan M. (2018). Research on properties of bio-asphalt binders based on time and frequency sweep test. Constr. Build. Mater..

[37] Zhang H., Chen Z., Xu G., Shi C. (2018). Evaluation of aging behaviors of asphalt binders through different rheological indices. Fuel.

[38] Cheng L., Yu J., Zhao Q., Wu J., Zhang L. (2020). Chemical, rheological and aging characteristic properties of Xinjiang rock asphalt-modified bitumen. Constr. Build. Mater..

[39] ASTM-D7175-15 (2017). Standard Test Method for Determining the Rheological Properties of Asphalt Binder Using a Dynamic Shear Rheometer 1. Annu. B Stand.

[40] Chailleux E., Ramond G., Such C., de La Roche C. (2006). A mathematical-based master-curve construction method applied to complex modulus of bituminous materials. Road Mater. Pavement Des..

[41] Dondi G., Vignali V., Pettinari M., Mazzotta F., Simone A., Sangiorgi C. (2014). Modeling the DSR complex shear modulus of asphalt binder using 3D discrete element approach. Constr. Build. Mater..

[42] Booshehrian A., Mogawer W.S., Bonaquist R. (2013). How to Construct an Asphalt Binder Master Curve and Assess the Degree of Blending between RAP and Virgin Binders. J. Mater. Civ. Eng..

[43] Gonzalez-Torre I., Norambuena-Contreras J. (2020). Recent advances on self-healing of bituminous materials by the action of encapsulated rejuvenators. Constr. Build. Mater..

[44] Wu S., Ye Y., Li Y., Li C., Song W., Li H., Li C., Shu B., Nie S. (2019). The effect of UV irradiation on the chemical structure, mechanical and self-healing properties of asphalt mixture. Materials.

[45] Jing R., Varveri A., Liu X., Scarpas A., Erkens S. (2019). Laboratory and Field Aging Effect on Bitumen Chemistry and Rheology in Porous Asphalt Mixture. Transp. Res. Rec. J. Transp. Res. Board.

[46] Zeng W., Wu S., Wen J., Chen Z. (2015). The temperature effects in aging index of asphalt during UV aging process. Constr. Build. Mater..

[47] Yuhong Wang P.E., Wen Y., Zhao K., Chong D., Wong A.S.T. (2014). Evolution and locational variation of asphalt binder aging in long-life hot-mix asphalt pavements. Constr. Build. Mater..

[48] Wu J. (2009). The Influence of Mineral Aggregates and Binder. http://awww.ukdctn.org/research/groups/ntec/documents/theses/wu,jiantao(jed)thesis15-6-09.pdf.

[49] Yang Z., Zhang X., Zhang Z., Zou B., Zhu Z., Lu G., Xu W., Yu J., Yu H. (2018). Effect of aging on chemical and rheological properties of bitumen. Polymers.

[50] Qin Q., Schabron J.F., Boysen R.B., Farrar M.J. (2014). Field aging effect on chemistry and rheology of asphalt binders and rheological predictions for field aging. Fuel.

[51] Petersen J.C., Glaser R. (2011). Asphalt oxidation mechanisms and the role of oxidation products on age hardening revisited. Road Mater. Pavement Des..

[52] Petersen J.C. (2009). A Review of the Fundamentals of Asphalt Oxidation: Chemical, Physicochemical, Physical Property, and Durability Relationships. https://trid.trb.org/view/902386.

